# XEN Gel Stent for Conjunctiva with Minimal Mobility Caused by Scleral Encircling: A Case Report

**DOI:** 10.3390/jcm12134293

**Published:** 2023-06-27

**Authors:** Yuri Kim, Myungjin Kim, Dai Woo Kim, Seungsoo Rho

**Affiliations:** 1 Department of Ophthalmology, Bundang CHA Medical Center, CHA University, Seongnam 13496, Republic of Korea; kimyuri0550@gmail.com (Y.K.); brandmjeyes@gmail.com (M.K.); 2 Department of Ophthalmology, School of Medicine, Kyungpook National University, Daegu 41944, Republic of Korea; proector97@gmail.com

**Keywords:** MIGS, XEN gel stent, scleral buckling, blebs

## Abstract

This case report describes the successful use of a XEN gel stent for controlling intraocular pressure (IOP) in a patient who had previously undergone scleral encircling for rhegmatogenous retinal detachment. The patient had very limited mobile conjunctiva due to scarring caused by the earlier surgery, which limited their options for glaucoma surgery. The XEN gel stent, a minimally invasive glaucoma surgery (MIGS) procedure that does not require opening the conjunctiva, was implanted in the subconjunctival space using an ab interno approach. Postoperative blebs were imaged using anterior segment optical coherence tomography, and IOP was monitored over six months. This study found that the XEN gel stent effectively controlled the IOP, and there were no complications during or after surgery. This case report may expand the indication for the XEN gel stent, which could be considered a viable option for patients who have undergone scleral buckling and have limited mobile conjunctiva.

## 1. Introduction

Minimally invasive glaucoma surgery (MIGS) represents a collection of conjunctival-sparing, ab interno procedures to control intraocular pressure (IOP) in primary open-angle glaucoma or pseudoexfoliation glaucoma eyes [1]. In particular, the bleb-forming glaucoma procedure necessitates the use of conjunctiva; therefore, the more mobile the conjunctiva, the better the outcome. Rhegmatogenous retinal detachment is the most common retinological emergency threatening vision, with an incidence of 1 in 10,000 persons per year, which can cause blindness in the affected eye without proper treatment [2,3]. However, there is no valid justification for refraining from conducting retinal detachment surgery solely based on the presence of mobile conjunctiva, which may potentially be involved in glaucoma surgery at a later point in time. In particular, scleral buckling causes scarring due to damage to the conjunctiva and creates more fibrotic conjunctiva with less mobile area, which reduces the selection width of glaucoma surgical options.

We would like to report a case that successfully formed blebs using a XEN gel stent through the least mobile conjunctiva remaining in the eyes owing to previous scleral buckling. To the best of our knowledge, this is the first report that describes that a XEN gel stent implantation using an ab interno approach can control the IOP in an eye with previous scleral buckling history. This report also offers the morphologic evaluation of the bleb for six months using anterior segment optical coherence tomography (AS-OCT) and might help endeavors to expand the indication for the XEN gel stent in eyes with minimally mobile conjunctiva.

## 2. Case Presentation

**Figure 1:**
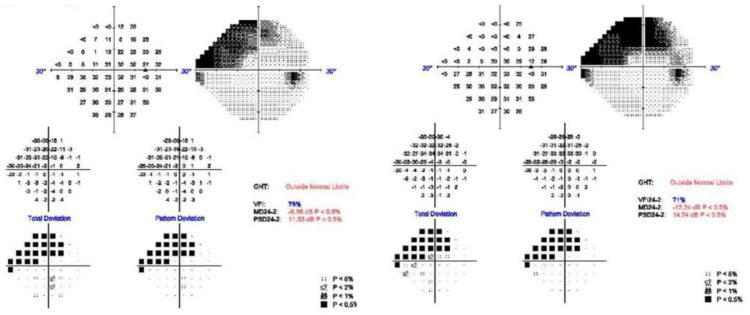
Visual field change before XEN gel stent implantation. Note that the visual field index decreased from 79% (left) to 71% (right) in the last year before the decision for surgery.

A 49-year-old female was referred to glaucoma service for IOP control. She first visited our hospital for further surgical management of the right eye fourteen years ago. Due to rhegmatogenous retinal detachment, the eye underwent scleral encircling with a 360-degree conjunctiva peritomy using a 4 mm thick sponge, cryopexy (performed between 10:30 and 1:30 o’clock), and pars plana vitrectomy. After one month, the silicone sponge was removed due to uncontrolled IOP. IOP normalized after the sponge was removed. After five years, the patient had undergone cataract surgery in the same eye. The IOP was around 20 mmHg despite maximally tolerated medical treatment, which included preservative-free dorzolamide and timolol, preservative-free 0.15% brimonidine tartrate, and preservative-free latanoprost eye drops. At the time of presentation to glaucoma service, which was thirteen years after the encircling, her best-corrected visual acuity in the right eye remained 20/20, but the IOP was 25 mmHg. The axial length was 26.53 mm, which implies a highly myopic eye. One year after, the visual field index fell from 79% to 71% in the last year (Figure 1). Apart from the uncontrolled IOP and progressing visual field deterioration, surgical intervention was considered, since her eye became allergic and she complained of eyelid change to brimonidine and bimatoprost eye drops, respectively. As possible surgical options, an Ahmed glaucoma valve, trabeculectomy, or a XEN gel stent were prepared. The surgeon had a scrutinized preoperative interview with the patient on the best, most possible option, which would be decided according to the result of the conjunctival mobility check using an injection of air, lidocaine, etc.

**Figure 2:**
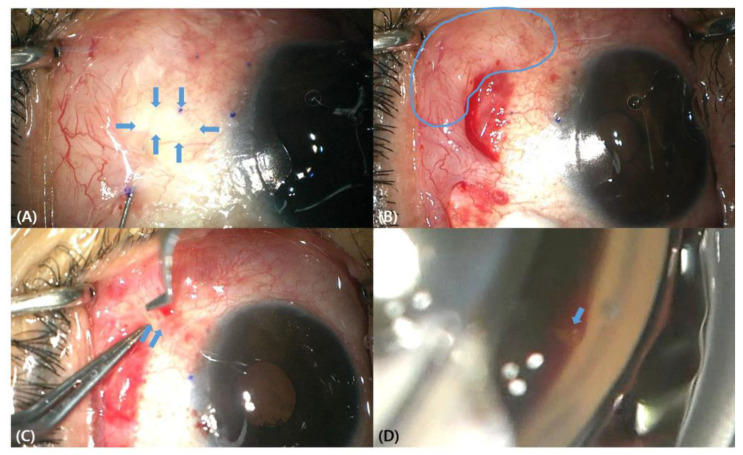
Intraoperative findings for XEN gel stent under a surgical microscope. (**A**) Blue arrows indicate immobile conjunctiva due to adhesion. (**B**) Blue line indicates spared conjunctiva. (**C**) Blue arrows indicate XEN gel stent. (**D**) Blue arrow indicates XEN gel stent during gonioscopy examination.

Surgical procedures were performed by a skilled surgeon (S.R.) as described elsewhere [4]. Briefly, after topical anesthesia, air and ocular viscoelastics were injected into subconjunctival space to dissect the conjunctiva and the tenon’s capsule to confirm the presence of the mobile conjunctiva. Almost the entire conjunctiva was immobile due to adhesion (Figure 2A), but fortunately, some spared mobile conjunctiva was noted (Figure 2B) in the superonasal quadrant. A 0.05 mL mix of 2% lidocaine with epinephrine (1:10,000, 0.1 mL) was injected using a 30 G needle into the superior subconjunctival space located approximately 6 mm apart from the region that the XEN tip is expected to occupy. After an injection of viscoelastics to maintain the anterior chamber using a 1 mm side port, the XEN injector was advanced through a 1.5 mm clear corneal incision at the inferotemporal limbus toward the opposite superonasal target angle The injector was approached to the angle into the subconjunctival space 2 mm apart from the limbus. After confirming the allocation of the XEN gel stent in the mobile conjunctiva by the dissection described earlier, the injector was moved backwards and removed gently out of the corneal incision. The proper location and length (approximately 1 mm) of the stent in the anterior chamber were checked using a surgical gonioscope, and the mobility and length (approximately 3 mm) of the subconjunctivally located part of the stent were confirmed (Figure 2C,D). Irrigation and aspiration were carried out to remove the viscoelastics. The corneal wounds were secured by hydrosealing using a balanced salt solution. A quantity of 0.05 mL mitomycin C (MMC) 0.4 mg/mL was injected into the superonasal subconjunctival space using a 30 G needle [4].

**Figure 3:**
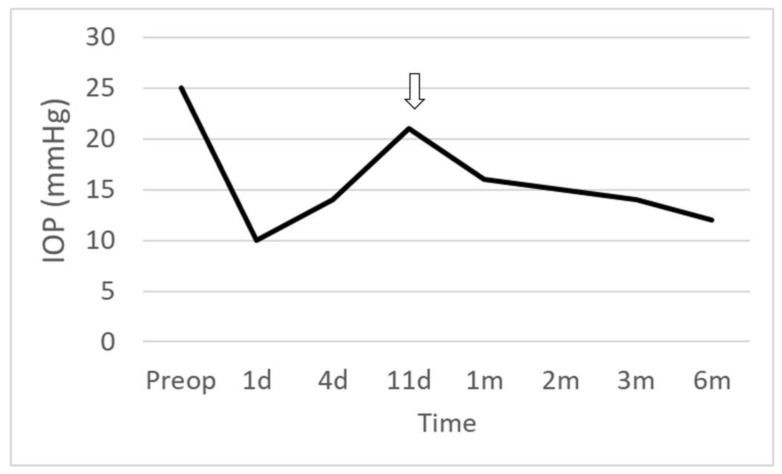
The change in intraocular pressure (IOP). Note that the IOP rose to 21 mmHg on postoperative day 11 (*white arrow*). The IOP was controlled after the readministration of timolol/dorzolamide eye drops once daily.

**Figure 4:**
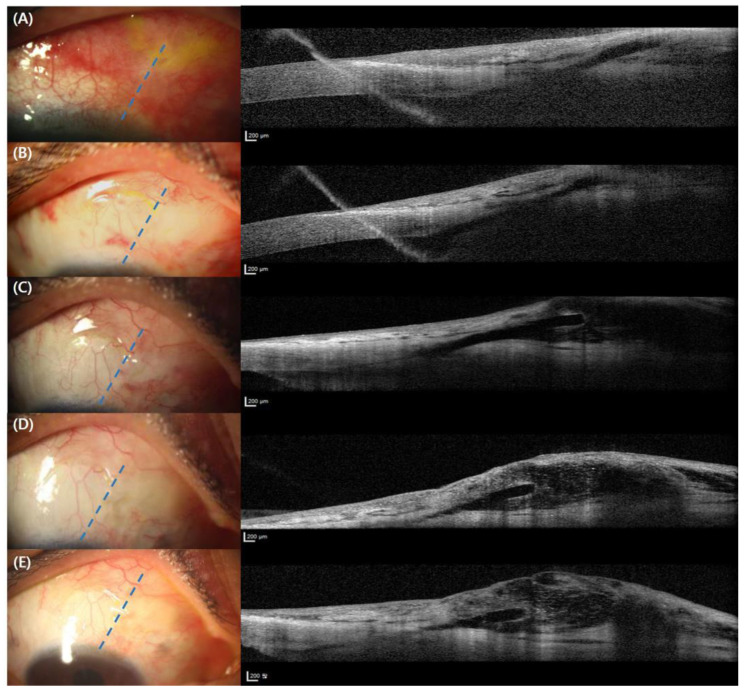
Bleb morphology using slit-lamp photos and AS-OCT imaging at postoperative day 1 (**A**), day 4 (**B**), day 14 (**C**), month 1 (**D**), month 3 (**E**), respectively. Note that the bleb morphology observed using slit-lamp photography exhibits characteristics of a localized avascular nature, while the bleb morphology observed using anterior segment optical coherence tomography (AS-OCT) demonstrates characteristics of a high sparse wall type. Please note that blue dashed lines represent the same location of each OCT section.

On the first day following surgery, the IOP was 10 mmHg, and moxifloxacin, prednisolone, and mydriatic eye drops were administered 4 times a day. On the 4th day after surgery, the IOP was 14 mmHg, and the vision had fully recovered to the preoperative level of 20/20. On the 11th day after surgery, the IOP was 21 mmHg, so once-daily use of preservative-free timolol/dorzolamide eye drops was initiated, and the IOP was maintained at 16 → 14 → 12 mmHg (1 → 3 → 6 months after surgery, Figure 3). Using slit-lamp examination of the bleb morphology, a localized avascular bleb was observed. Checking the morphology of blebs using OCT, a high, sparse wall was observed during the all follow-ups (Figure 4). The lengths of the fluid-filled cavity parallel and perpendicular to the section line at 6 months were 3.065 mm and 3.555 mm. The estimated size of the bleb was 34.21 mm^2^.

This study adhered to the principles of the Declaration of Helsinki. Written informed consent for the report and photographs was obtained from the patient. Postoperative blebs were imaged using slit-lamp photography and a Spectralis OCT (Heidelberg Engineering GmbH, Heidelberg, Germany) on postoperative day 1, week 1, week 2, and months 1, 2, 3, and 6, as described elsewhere. Briefly, a total of 41 section scans were aligned along with the parallel line at the point where the XEN tip was located. Images with quality scores higher than 25 were included in the final qualitative analysis. The maximum bleb height was selected and measured within the 41 sections that were obtained at each visit [4].

## 3. Discussion

The XEN gel stent is recognized as a safe and effective MIGS procedure compared to conventional trabeculectomy [5]. Both filtration surgeries are considered bleb-forming surgery, which depends on the size of their blebs. A healthy conjunctiva is required for both bleb-forming glaucoma operations. Watanabe-Kitamura et al. reported that, at 12 months post-trabeculectomy, the volume ratio correlated with the IOP and the number of eye drops needed using a 3D investigation of bleb volume after trabeculectomy. [6] Kawana et al. evaluated that the IOP showed a significant negative correlation with the horizontal and vertical lengths of the fluid-filled cavity, the height of the cavity, and the volume of the internal fluid-filled cavity [7]. The mean horizontal and vertical lengths of the cavity of successful blebs were longer than those of failed blebs (3.86 ± 2.16 mm and 4.23 ± 2.06 mm vs. 1.21 ± 1.05 mm and 1.39 ± 1.16 mm, respectively). The length of the internal cavity, in our case, also lies in this range (3.065 mm), which could imply the existence of a minimum level of bleb size. Although the IOP control after glaucoma filtration surgery is known to be influenced by the morphology and the size of the bleb after trabeculectomy [8], the minimum size of the bleb for the proper IOP control is expected. In this vein, the addition of our report can offer possible inspiration for further research on this matter.

Other quantitative parameters of the bleb associated with internal bleb morphology are also related to the IOP control. The IOP showed a negative correlation with the number of microcysts and with the volume of the hyporeflective area [7]. The bleb vascularity score at 12 months was related to the volume ratio of the bleb wall [6]. In a previous study, we compared the mean IOPs according to the bleb classifications in AS-OCT and slit-lamp images using post hoc analysis. A diffuse avascular bleb showed a lower mean IOP compared with a localized vascular bleb with statistical significance. Moreover, a localized avascular bleb showed a relatively lower mean IOP compared with a localized vascular bleb, although it did not reach statistical significance [5]. The bleb in our study can be classified as a localized avascular type, which implies that the IOP control could be better than vascular blebs.

Scleral buckling procedures can challenge IOP control in glaucoma eyes by compressing vortex veins, causing increased episcleral venous pressure [9]. Moreover, dissection of the conjunctiva inevitably induces conjunctival scarring, which could limit the feasibility of glaucoma filtration surgery. Since trabeculectomy necessitates enough healthy conjunctiva, glaucoma drainage device implantation has traditionally been preferred over trabeculectomy in glaucoma eyes with previous scleral buckling history. Therefore, it is important to preserve mobile conjunctiva as much as possible to minimize conjunctival scarring for these eyes. Vu and Junk reported that ab externo XEN gel stent implantation may successfully reduce IOP without interfering with the previous scleral buckle for three months postoperation [9]. However, the ab externo approach needs additional conjunctival dissection, which causes more fibrosis, reducing the chance of further manipulation of the surgical area in the future. In contrast, the ab interno approach method is generally considered the best way to achieve minimal invasiveness.

Surgical options for managing glaucoma eyes with minimally mobile conjunctiva are limited and challenging. Due to the previous history of encircling, superotemporal and superonasal quadrants for conjunctival dissections in both a trabeculectomy and a tube were not feasible in our cases. The original definition of MIGS tends to encompass any procedure that avoids conjunctival dissection and is approached using an ab interno clear corneal incision [1]. If the superonasal bleb created by the first XEN fails, the inferonasal quadrant can be considered the next target. Moreover, endoscopic cyclophotocoagulation (ECP) is one of the few MIGS procedures that aim to lower IOP in glaucoma eyes with minimally mobile conjunctiva where other MIGS procedures are all contraindicated. Rodrigues et al. reported that ECP can be safely and successfully performed to control IOP in an eye with severe scleromalacia [10]. However, ECP is not available in the South Korean market. Ab interno XEN gel stent implantation and endoscopic cyclophotocoagulation have the benefit of offering an exit strategy by not precluding any further glaucoma surgery, which is supposed to be one of the major advantages of MIGS.

## 4. Conclusions

We reported a case of refractory glaucoma with minimally mobile conjunctiva, which was successfully managed using one of the widely performed MIGS procedures, XEN gel stent implantation. A XEN gel stent is a good option for controlling IOP in glaucoma following scleral buckling surgery, especially using the ab interno approach. This is the first case in which a XEN gel stent was implanted using an ab interno approach and is feasible for controlling IOP where there is limited healthy mobile conjunctiva space.

## Data Availability

The data presented in this study are available on request from the corresponding author. The data are not publicly available due to privacy.
